# Peroxide Responsive Regulator PerR of group A Streptococcus Is Required for the Expression of Phage-Associated DNase Sda1 under Oxidative Stress

**DOI:** 10.1371/journal.pone.0081882

**Published:** 2013-12-03

**Authors:** Chih-Hung Wang, Chuan Chiang-Ni, Hsin-Tzu Kuo, Po-Xing Zheng, Chih-Cheng Tsou, Shuying Wang, Pei-Jane Tsai, Woei-Jer Chuang, Yee-Shin Lin, Ching-Chuan Liu, Jiunn-Jong Wu

**Affiliations:** 1 Departments of Medical Laboratory Science and Biotechnology, College of Medicine, National Cheng Kung University, Tainan, Taiwan; 2 Department of Microbiology and Immunology, College of Medicine, Chang Gung University, Taoyuan, Taiwan; 3 Institute of Basic Medical Science, College of Medicine, National Cheng Kung University, Tainan, Taiwan; 4 Departments of Microbiology and Immunology, College of Medicine, National Cheng Kung University, Tainan, Taiwan; 5 Departments of Biochemistry, College of Medicine, National Cheng Kung University, Tainan, Taiwan; 6 Departments of Pediatrics, College of Medicine, National Cheng Kung University, Tainan, Taiwan; 7 Center of Infectious Disease and Signaling Research, National Cheng Kung University, Tainan, Taiwan; University of South Dakota, United States of America

## Abstract

The peroxide regulator (PerR) is a ferric uptake repressor-like protein, which is involved in adaptation to oxidative stress and iron homeostasis in group A streptococcus. A *perR* mutant is attenuated in surviving in human blood, colonization of the pharynx, and resistance to phagocytic clearance, indicating that the PerR regulon affects both host environment adaptation and immune escape. Sda1 is a phage-associated DNase which promotes M1T1 group A streptococcus escaping from phagocytic cells by degrading DNA-based neutrophil extracellular traps. In the present study, we found that the expression of *sda1* is up-regulated under oxidative conditions in the wild-type strain but not in the *perR* mutant. A gel mobility shift assay showed that the recombinant PerR protein binds the *sda1* promoter. In addition, mutation of the conserved histidine residue in the metal binding site of PerR abolished *sda1* expression under hydrogen peroxide treatment conditions, suggesting that PerR is directly responsible for the *sda1* expression under oxidative stress. Our results reveal PerR-dependent *sda1* expression under oxidative stress, which may aid innate immune escape of group A streptococcus.

## Introduction

*Streptococcus pyogenes* (group A streptococcus, GAS) is a facultative Gram-positive human pathogen which causes mild to severe infectious diseases including pharyngitis, cellulitis, necrotizing fasciitis, and toxic shock syndrome [1,2]. Although it lacks catalase, GAS has developed other defense mechanisms against oxidative stress, including NADH oxidase, superoxide dismutase, peroxidases, and Dps-like peroxide resistance protein [3,4]. In addition, a peroxide operon regulator, PerR, has been identified and characterized as the peroxide responsive regulator in GAS [3,5,6].

PerR is a ferric uptake repressor (Fur)-like protein, which regulates genes involved in oxidative stress responses and iron homeostasis in GAS [3,6-8]. PerR protein has been shown to bind to zinc and iron, and the metal binding of PerR is required for optimal responses to peroxide [5,9]. The *perR* mutant is attenuated in virulence in murine air sac and baboon pharyngitis infection models [3,10]. In addition, the *perR* mutant is more susceptible to phagocytic cell clearance [10], indicating that the PerR regulon also contributes to immune escape. Transcriptome analysis further showed that PerR not only regulates peroxide detoxifying enzyme expression, but also coordinates DNA and protein metabolisms and DNA repair system activity, which may contribute to GAS virulence and adaptation to the host environment [5,11].

DNase is one of the important virulence factors of GAS [12,13]. Until now, DNases Sda1, Spd, Mf3, and SpnA have been identified in GAS [12,14-16]. Mf3 and Sda1 are encoded by prophages, but Spd and SpnA are suggested to be chromosomally encoded [17,18]. SpnA is the only cell-wall-anchored DNase found in GAS [15]. Sumby et al. (2005) showed that extracellular DNase activity is required for normal progression of GAS infection in mouse and non-human primate infection model. Among them, Sda1 is the major DNase that contributes to the virulence of the clinical relevant M1T1 clone [17,19]. Buchanan et al. (2006) showed that Sda1 is both necessary and sufficient to promote GAS virulence in a murine model of necrotizing fasciitis. Compared to the wild-type strain, the *sda1* mutant is impaired in its ability to degrade neutrophil extracellular traps (NETs), resulting in efficient clearance by neutrophils [12,19]. Furthermore, a recent study further showed that GAS employs Sda1 to prevent Toll-like receptor-9 recognition of degraded bacterial DNA [20]. These results indicate that Sda1 has important roles in GAS escaping immune clearance.

In the present study, we show that the increased expression of *sda1* under oxidative stress is PerR-dependent. In addition, mutation of the metal-binding site of PerR abolished its positive regulation of *sda1*, suggesting that the peroxide responsive activity of PerR is crucial for regulating *sda1* expression. These results suggest that PerR contributes to GAS immune escape through regulating *sda1* expression under oxidative conditions.

## Materials and Methods

### Bacterial strains and culture media

GAS strain A20 (*emm1*/ST28), SW612 (the *perR* isogenic mutant), and SW665 (*perR* complementation strain) were described in the previous study [21]. GAS strains were cultured in tryptic soy broth (Becton, Dickinson and Company, Sparks, MD) supplemented with 0.5% yeast extract (TSBY) at 37°C. *E. coli* DH5α or HB101 (Bethesda Research Laboratories, Gaithersburg, MD) were cultured in Luria-Bertani (LB) broth at 37°C with vigorous aeration.

### Bacterial Growth Curve Measurement

Bacterial growth curves were measured by the method described previously [22]. GAS strains were cultured in TSBY broth at 37°C for 12-16 h. One hundred µl of overnight culture was transferred to 10 ml of fresh TSBY broth and incubated at 37°C without agitation. The optical density of the bacterial suspension was measured by UV/visible spectrophotometer (GE Healthcare, Uppsala, Sweden) after 4-12 h incubation.

### DNA and RNA manipulation

GAS genomic DNA extraction, RNA extraction, and reverse transcription were described previously [23]. Real-time PCR reactions were performed in a 10 µl mixture containing 2 µl of cDNA, 0.3 µl of primers (10 µM), and 5 µl of KAPA SYBR^®^ Fast qPCR pre-mixture (KAPA Biosystems, Woburn, MA). The mixtures were incubated at 95°C for 10 min, followed by 45 cycles of 95°C (20 sec), 60°C (1 min), and 72°C (15 sec). Real-time quantitation was analyzed by LightCycler software (version 3.0, Roche Diagnostics, Indianapolis, IN). Expression level of each target gene was normalized to *gyrA* (Table 1) and analyzed using the ∆∆Ct method. Biological replicate experiments were performed from at least three independent RNA preparations in duplicate. Primers used for real-time PCR analysis were designed by Primer3 (v. 0.4.0, http://frodo.wi.mit.edu) according to the MGAS5005 chromosomal DNA sequence (NCBI reference sequence: NC_007297.1) and are described in Table 1. RNAs extracted from A20 (cultured in TSBY for 5 h) were used for 5’ rapid amplification of cDNA ends (RACE) analysis 5’ RACE analysis was performed according to methods described in the manual (5’ RACE System for Rapid Amplification of cDNA Ends, Version 2.0, Life Technologies Cooperation).

**Table 1:** Primers used in this study.

Target gene (locus tag)	Use	Forward primer	Reverse primer
*mf3* (M5005_Spy_1169)	Real-time PCR	Gcgactgagacaccaggaa	ttatgccagccaggagga
*spd* (M5005_Spy_1738)	Real-time PCR	Cacgacagctcttggaatca	cgttacagggacacgtaccc
*sda1* (M5005_Spy_1415)	Real-time PCR	Tcgtacaaccgaaagggtct	cttggctctggtttgctttc
*spnA* (M5005_Spy_0571)	Real-time PCR	Tgtggctaaagcagtgacca	gccactaacatgccttccat
*gyrA* (M5005_Spy_0874)	Real-time PCR	Cgtcgtttgactggtttgg	ggcgtgggttagcgtattta
P*sda1*	Construction	cgagctcgcaacacttcttccacttttt	cgggatcctatttatgtcctccttttgt
*perR* gene with His99Ala mutation	Construction	atgggccatcaagcagtcaatgtagtt	aactacattgactgcttgatggcccat

### Plasmid Constructions

Plasmid pMW508 used for mutated the *perR* gene in wild-type strain A20 was described previously [21]. Briefly, the 1.7 kb of *perR* region was amplified by PCR and cloned in to the plasmid pSF152. The internal region of the *perR* gene (196-468 bp) was deleted by the inverted PCR, and then the chloramphenicol resistance cassette was ligated into the deletion region. Plasmid pMW508 was electroporated into A20, and the *perR* isogenic mutant was confirmed by Southern hybridization with the Cm probe [21]. For complementation of the *perR* mutant, the 1.5 kb of DNA containing the *perR* gene and the promoter region were amplified by PCR and cloned in to the *E. coli*-*Streptococcus* shuttle vector pDL278 (copy number is around 20 to 30) [24]. For measurement of the *sda1* promoter activity, the *sda1* promoter (400 bp) was amplified and fused with a promoter-less *cat* gene on plasmid pMW398 [25] by using the *Bam*HI restriction enzyme site to construct plasmid pMW755. To mutate the histidine residue (H99) in the putative metal binding site of PerR [26], the nucleotide sequence (CAC) encoding for histidine (residue 99) was mutated to GCA (encoding for alanine) by site directed mutagenesis with primers described in Table 1. The mutated *perR* gene with its native promoter was ligated to pDL278 by *Bam*HI and *Eco*RI to construct plasmid pMW680.

### Gel mobility shift assay

Purification of recombinant His_6_-PerR protein (rPerR) and the gel mobility shift assay were described previously [21]. A DNA probe of the *sda1* promoter region containing the PerR-binding sequence was amplified by primer *sdaD2* promoter-1-SacI (CGAGC TCGCA ACACT TCTTC CACTT TTT) and *sdaD2* promoter-2-KpnI (GGGGT ACCCT ATTTA TGTCC TCCTT TTGT). The control DNA probe (P*dpr*) and a non-specific promoter DNA of *SPy1840* were amplified by primers described previously [21]. rPerR and 50 ng of DNA probes were incubated in binding buffer (20 mM of Tris-HCl pH 8.0, 5% glycerol, 50 µg/ml of bovine serum albumin, and 50 mM of KCl) at room temperature for 15 min. The reaction mixtures were analyzed with a 6% native polyacrylamide gel and DNA-protein complexes were visualized by staining with ethidium bromide. Competition assays were performed according to the manual (DIG Gel Shift Kit, Roche, Indianapolis, IN). Unlabeled DNA probes (cold probes) were amplified by primers described in Table 1.

### Statistical analysis

Statistical analysis was performed by using Prism software, version 4 (GraphPad, San Diego, CA). A P value of student’s *t* test < 0.05 was taken as significant.

## Results

### Expression of DNase genes in the *perR* mutant

**Figure 1:**
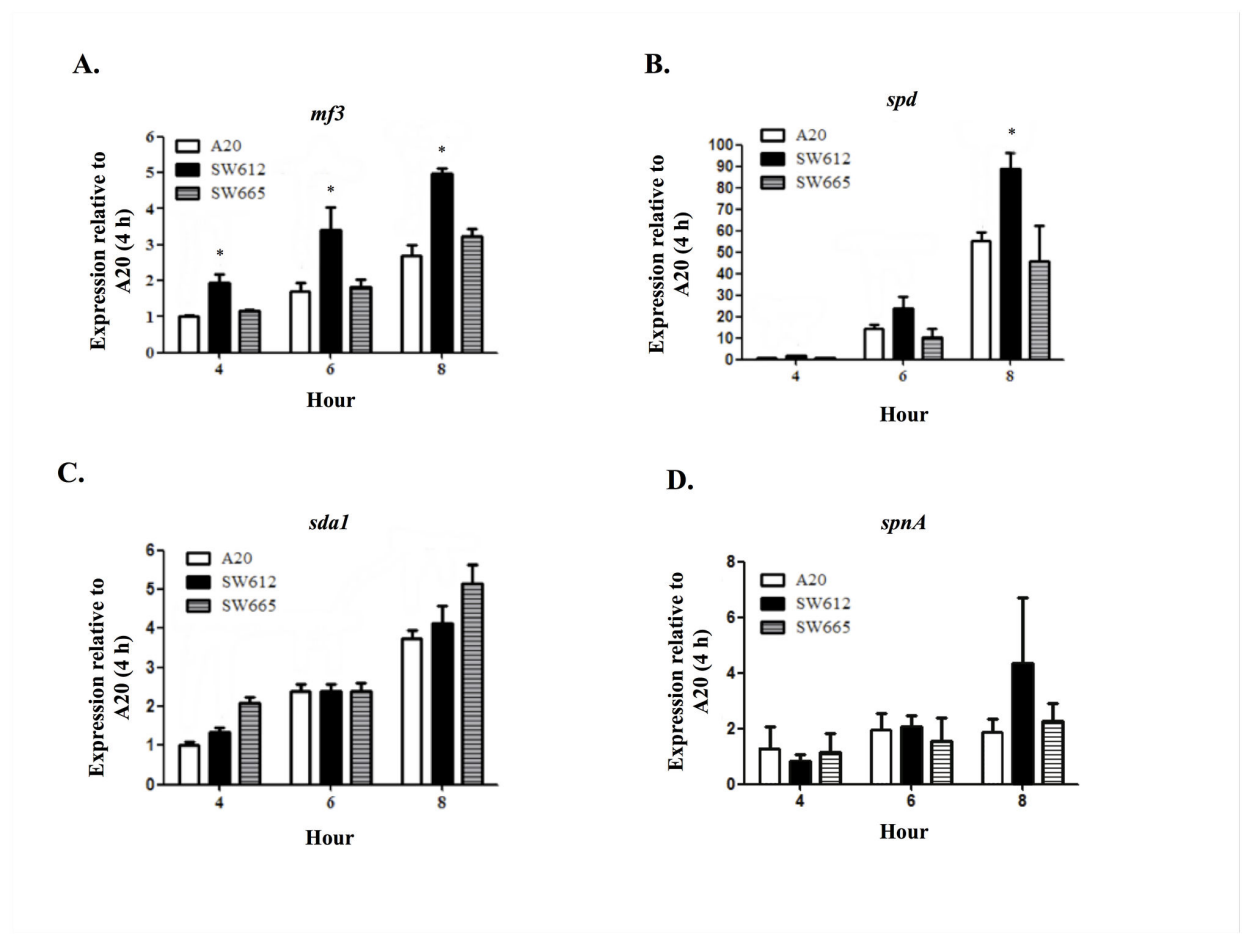
Expression of DNase genes in the wild-type (A20), *perR* mutant (SW612), and complementation strain (SW665) under normal culture conditions. Bacteria were cultured in TSBY broth and were collected for RNA extraction after 4, 6, and 8 h incubation. RNAs (from at least three independent RNA extracts) were converted to cDNA and the expression of DNase genes including (A) *mf3*, (B) *spd*, (C) *sda1*, and (D) *spnA* in A20, SW612, and SW665 were analyzed by real-time PCR. Expression of target genes was normalized to *gyrA*. *: *P* < 0.05 to both A20 and SW665 at each time point.

A *perR* mutant is more resistant to oxidative stresses *in vitro*, but more susceptible to phagocytic cell clearance when compared to the wild-type strain [3,10]. DNases of GAS have been reported to have important roles in escaping immune clearance by degrading the DNA of the neutrophil extracellular traps [12,13]. The roles of PerR in regulating expression of DNase genes were therefore analyzed. The wild-type (A20), *perR* mutant (SW612), and complementation (SW665) strains were cultured in TSBY broth. No significant difference was found in the growth of these strains (Figure S1). The expression of *mf3*, *spd*, *spnA*, and *sda1* in A20, SW612, and SW665 was analyzed by real-time RT-PCR. Results showed that the expression of *mf3* and *spd* are significantly increased in SW612 when compared to A20 and SW665 (Figure 1A and 1B). However, the expression of *sda1* and *spnA* showed no difference among these strains (Figure 1C and 1D).

### Expression of the DNase *sda1* under Oxidative Stress Is PerR-Dependent

**Figure 2:**
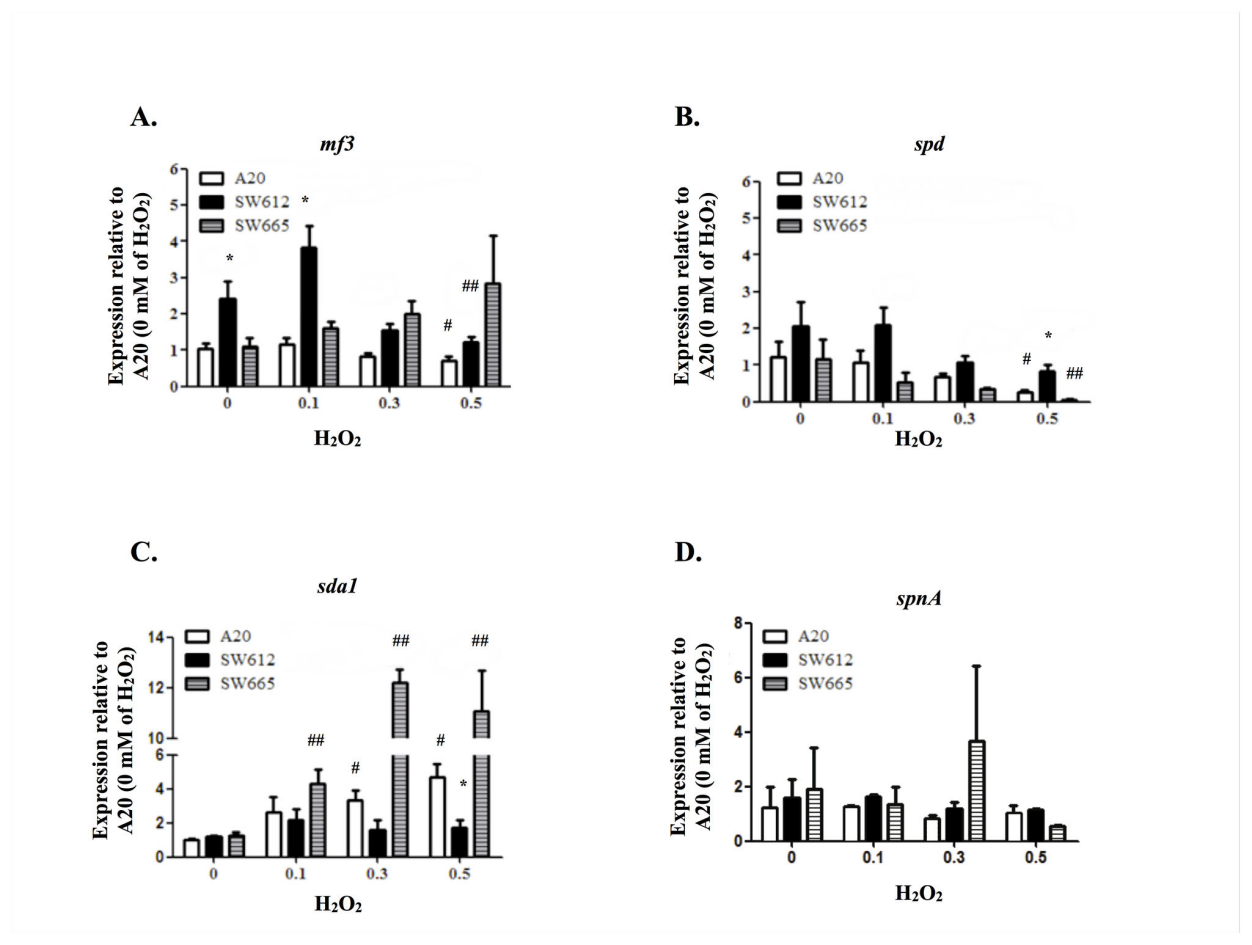
Expression of DNase genes in wild-type (A20), *perR* mutant (SW612), and complementation strain (SW665) under oxidative conditions. Bacteria were grown in TSBY broth for 3 h and treated with 0.1, 0.3, and 0.5 mM of H_2_O_2_ for another 2 h. The expression of DNase genes, including (A) *mf3*, (B) *spd*, (C) *sda1* and (D) *spnA*, were analyzed by real-time RT-PCR. Biological replicate experiments were performed from at least three independent RNA preparations in duplicate. Expression level of each target gene was normalized to *gyrA*. *: *P* < 0.05 to both A20 and SW665 in each culture condition; # and ##: *P* < 0.05 to A20 and SW665 without H_2_O_2_ treatment, respectively.

PerR is a peroxide responsive regulator in GAS [5,6,9,27]. The expression of DNase genes in the wild-type (A20), *perR* mutant (SW612), and complementation (SW665) strains were therefore analyzed under oxidative conditions. SW612 expresses more *mf3* under normal conditions and 0.1 mM hydrogen peroxide treatment when compared to A20 and SW665 (Figure 2A). However, the expression of *mf3* in A20 and SW612 were both significantly decreased after 0.5 mM hydrogen peroxide treatment (Figure 2A). The *spd* expression of A20 and SW665 was decreased in the presence of 0.5 mM of hydrogen peroxide, but showed no significant changes in SW612 (Figure 2B). Furthermore, the expression of *sda1* in A20 and SW665, but not SW612, was significantly increased after hydrogen peroxide treatment (Figure 2C). The *spnA* expression of A20 and SW612 showed no significant difference in the presence of or without hydrogen peroxide (Figure 2D).

### Determination of the transcriptional start site and putative Per box

**Figure 3:**
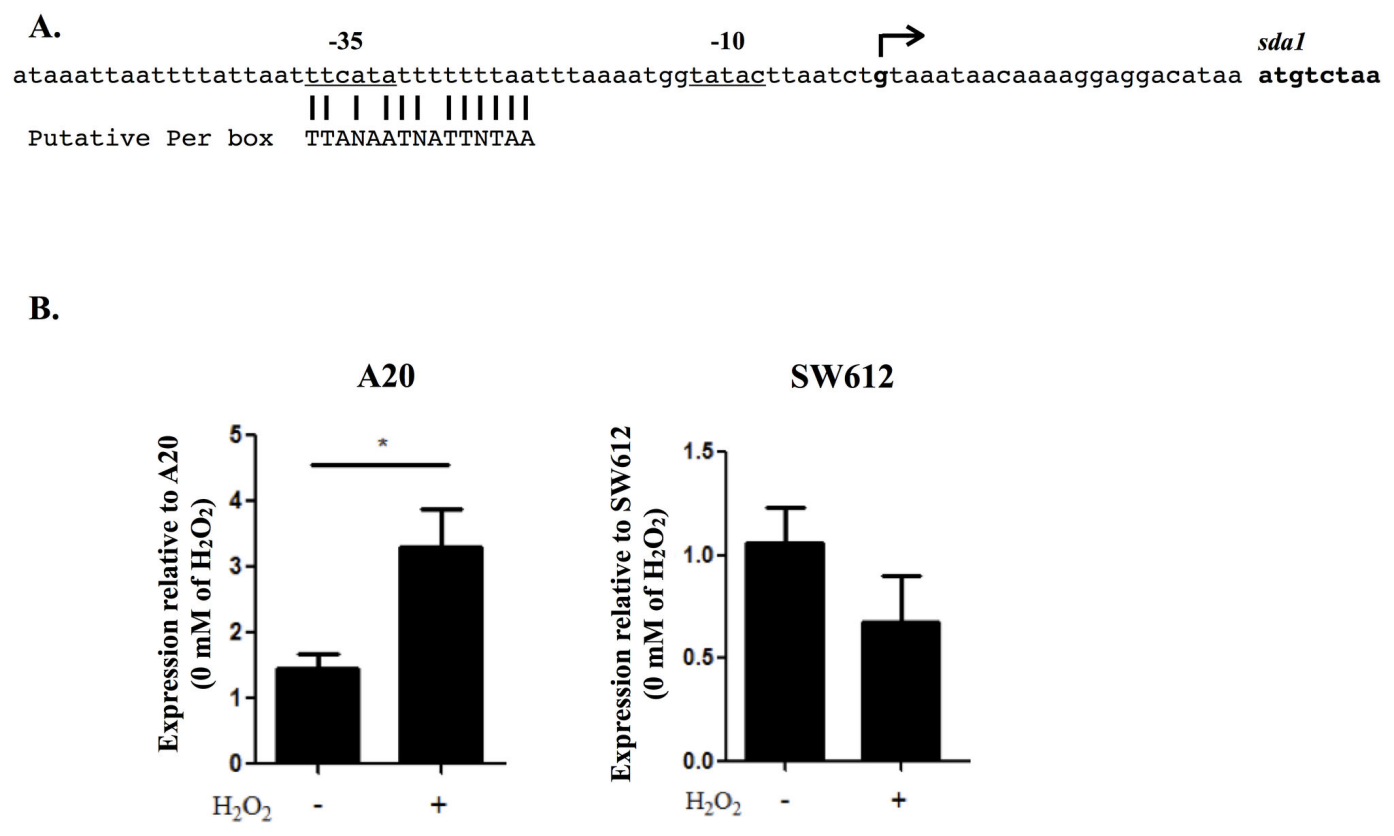
Localization of the putative Per box in the *sda1* promoter and the *sda1* promoter activity in the wild-type strain (A20) and *perR* mutant (SW612) in the presence or absence of hydrogen peroxide. (A) The putative Per box sequence in the *sda1* promoter region. The transcriptional start site of the *sda1* was determined by the 5’-RACE (indicates by arrow). (B) The *sda1* promoter activity in the wild-type strain (A20) and *perR* mutant (SW612). The reporter gene cat was fused with the wild-type *sda1* promoter (P*sda1*) and plasmid was transformed into A20 and SW612. The cat gene expression in A20 and SW612 in the presence or absence of 0.5 mM of H_2_O_2_ treatment was analyzed by real-time RT-PCR. Biological replicate experiments were performed from at least three independent RNA preparations in duplicate. Expression level of each target gene was normalized to *gyrA*. *: *P* < 0.05.

The observation that PerR regulates the *sda1* expression under oxidative stress conditions suggests that PerR may regulate *sda1* through the putative PerR-binding sequence (Per box) [7]. The transcriptional start site of *sda1* was determined by 5’-RACE assay (Figure 3A, indicates by arrow), and a putative Per box was found near the -35 region (Figure 3A). To further verify that the *sda1* transcription is respond to hydrogen peroxide treatments, the activity of the *sda1* promoter in the wild-type strain and *perR* mutant in the presence or absence of H_2_O_2_, were analyzed. Results showed that, in consistent with the *sda1* RNA expression (Figure 2C), the promoter activity of *sda1*, which was evaluated by the expression of the reporter gene *cat* (described in Materials and Methods), was only increased in the wild-type (A20) but not in the *perR* mutant (SW612) after hydrogen peroxide treatment (Figure 3B). 

### Recombinant PerR protein binds to the *sda1* promoter region

**Figure 4:**
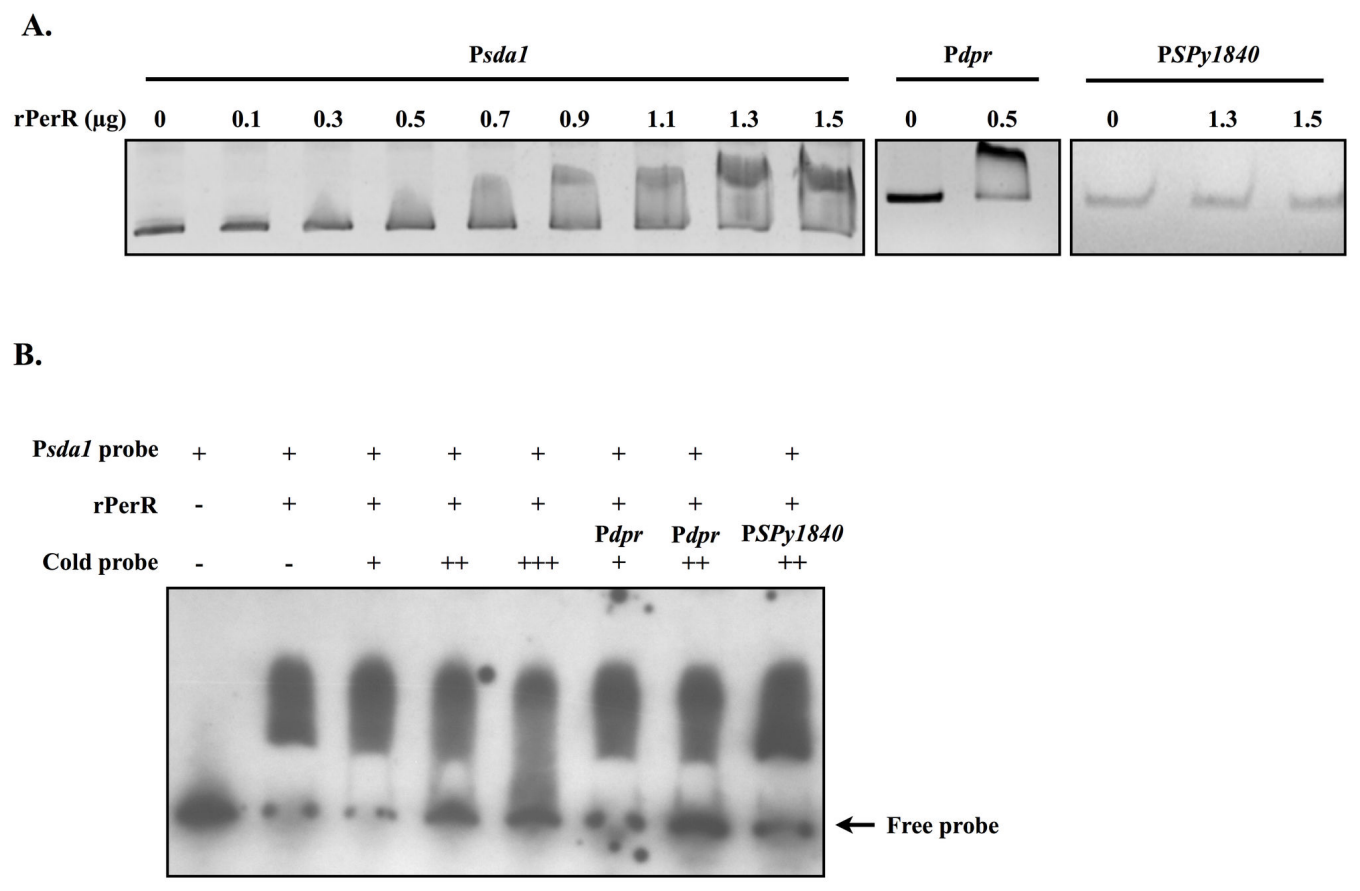
Interaction between rPerR and the *sda1* promoter. (A) DNA-binding activity of rPerR to different regions of the *sda1* promoter. rPerR protein was incubated with 50 ng of DNA probes (P*dpr* and P*SPy1840* as positive and negative control, respectively) and the DNA-binding activity was evaluated by the mobility shift of the protein-DNA complex in a 6% polyacrylamide gel. (B) Binding specificity of rPerR to P*sda1* DNA probe. DIG-labeled P*sda1* DNA probe was incubated with cold-specific probe (500, 1000, and 1500-fold of unlabeled P*sda1* DNA probe, and 500 and 1000-fold of P*dpr* DNA probe) or cold-nonspecific probe (P*spy1840* DNA probe). The protein-DNA complex in a 6% polyacrylamide gel was transferred to a membrane for visualizing the DIG signal.

To further clarify whether PerR bound to P*sda1*, the recombinant PerR protein (rPerR) was incubated with partial P*sda1* and the DNA binding activity of rPerR was analyzed by a gel retardation assay. Results showed that rPerR bound to the *dpr* promoter region (containing a PerR-binding box) but not to the *SPy1840* promoter (P*SPy1840*, without a PerR-binding box, Figure 4A). In addition, rPerR caused a band-shift of P*sda1* in a dose dependent manner (Figure 4A). To further investigate the specificity of binding, five hundred to one thousand-fold of unlabeled cold-specific probes (P*sda1* and P*dpr* DNA probes) and cold-nonspecific probe (P*SPy1840*) were used to compete against the protein-DNA interaction. Results showed that unlabeled P*sda1* probe can compete with the rPerR-P*sda1* interaction in a dose-dependent manner (Figure 4B). In addition, the cold-specific probe (P*dpr* probe) but not a cold-nonspecific probe (P*SPy1840*) significantly competes with the rPerR-P*sda1* interaction (Figure 4B).

### Metal binding site of PerR is required for regulating *sda1* under oxidative conditions

**Figure 5:**
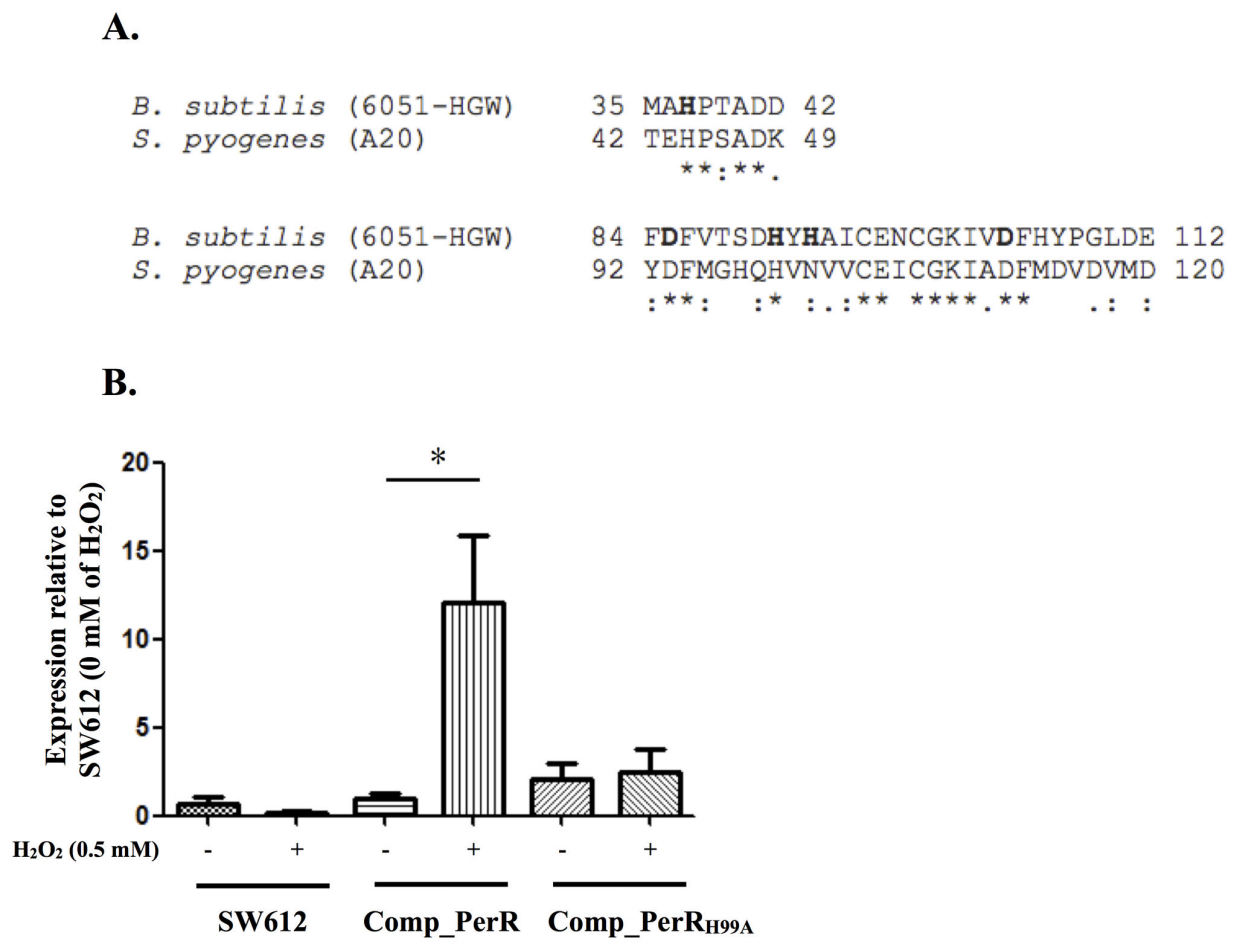
Expression of *sda1* in a *perR* mutant (SW612) complemented with the wild-type *perR* (Comp_PerR) or regulatory metal-binding site mutated *perR* (Comp_PerR_H99A_) under normal and H_2_O_2_ treatment conditions. Bacteria were grown in TSBY broth for 3 h and treated with/without 0.5 mM of H_2_O_2_ for another 2 h. The expression of *sda1* in different strains was analyzed by real-time RT-PCR. (A) Alignment of B. subtilis and S. pyogenes PerR protein sequence. Amino acid residues involved in metal binding in B. subtilis are shown in bold. (B) Expression of *sda1* in SW612, Comp_PerR, and Comp_PerR_H99A_ under normal and H_2_O_2_ treatment conditions. Biological replicate experiments were performed from at least three independent RNA preparations in duplicate. Expression level of each target gene was normalized to *gyrA*. *: *P* < 0.05.

Structural analysis of *B. subtilis* PerR (BsPerR) showed that BsPerR binds to a ferrous ion by the regulatory site, which is coordinated by three histidine (H37, H91, H93) and two aspartate (D85, D104) residues (Figure 5A) [26,28]. Under oxidative conditions, residues H37 and H91 of BsPerR are oxidized, resulting in altering PerR protein structure and its DNA-binding activity [26,27]. A recent study showed that mutation of histidine residues (H6, H19, and H99) of the regulatory site in GAS PerR reduces the metal occupancy but not affect DNA-binding ability of the PerR protein [9]. Peroxide sensing by PerR in GAS also requires regulatory metal [9]. To further clarify that whether the peroxide sensing ability of PerR of GAS is required for regulating *sda1* under oxidative conditions, the histidine residue (H99) of regulatory site in GAS PerR was mutated to alanine (PerR_H99A_, Figure 5A), and the expression of *sda1* in the *perR* mutant (SW612), and SW612 complemented with the wild-type PerR or PerR_H99A_ under normal and hydrogen peroxide treatment conditions were analyzed by real-time RT-PCR. Results showed that the expression of *sda1* in both SW612 and the PerR_H99A_ complemented strain were not responsive to the hydrogen peroxide treatment (Figure 5B). However, the increased expression of *sda1* under oxidative conditions was found in the wild-type PerR complementation strain (Comp_PerR, Figure 5B).

## Discussion

The peroxide response regulator (PerR) of GAS is important for bacterial survival in human blood, colonization of the pharynx, and resistance to phagocytic clearance, indicating that the PerR regulon is crucial for GAS to establish successful infections [3,10,11]. In the present study, we showed that PerR is required for the expression of phage-associated DNase *sda1* under oxidative conditions. Sda1 is a DNase of GAS and has roles in GAS escaping from immune recognition and clearance. The interaction between PerR and Sda1 may have important roles in GAS pathogenesis.

PerR is a metal-binding protein [27]. In *Bacillus subtilis*, a ferrous ion in the regulatory site of the PerR protein is involved in PerR protein conformation changes and DNA-binding activity under oxidative conditions [26,29]. In GAS, previous studies showed that PerR protein could bind to iron or manganese, and the DNA-binding activity of PerR is regulated by oxidative stress in vitro [5,9,21,30]. In the present study, EMSA analysis showed that rPerR binds to the P*sda1* promoter region, suggesting PerR may directly interact with the putative Per box of the *sda1* promoter (Figure 3). In addition, mutation of the histidine residue (H99) in the putative PerR regulatory site abolishes *sda1* expression under oxidative conditions (Figure 5), suggesting that oxidative stress-sensing ability of PerR is crucial for *sda1* expression. PerR generally is a repressor through directly binding to a target genes’ promoter [31,32]. Under oxidative conditions, the oxidized cations trigger the conformational changes in PerR, which involves decreasing PerR DNA-binding activity and derepression of target gene expression [27]. Although our results showed that PerR positively regulates *sda1* expression under oxidative conditions in GAS, the molecular mechanism of this positive regulation is still unknown. In *B. subtilis*, PerR positively regulates *srfA* gene (encodes for surfactin) and is required for its expression [32]. Although the mechanism by which PerR activates *srfA* remains unclear, Hayashi et al (2005) suggested that PerR may interact with other regulator to induce the full activation of *srfA* expression [32]. In GAS, according to the transcriptome analysis, Grifantini et al (2011) suggests that the oxidation PerR may not lead to its release from bound promoters in vivo [5]. In addition, protein structure analysis showed that GAS PerR possesses distinctive structural and amino acid features when compared to other Fur family proteins [9], suggesting that the molecular mechanism of PerR in GAS may be unique. Further studies of the interaction between PerR and the *sda1* promoter may provide clues for understanding the mechanism of this positive transcriptional regulation in GAS.

Transcriptome analysis showed that PerR in GAS not only involves regulating ROS-detoxifying enzymes, but also coordinates DNA and protein metabolic functions, and DNA repair system activity, which may contribute to GAS survival in the host environment [5,11,30]. Adapting to the host environment and escaping from immune clearance are required for bacteria to establish successful infections. Previous studies showed that a *perR* mutant is more susceptible to phagocytic cell clearance [10], indicating that the PerR regulon participates in immune escape of GAS. In the present study, we found that PerR may involve in repression of *spd* expression under oxidative stress (Figure 2B), but the putative Per box could not be found in the *spd* promoter region. Under oxidative stress, the expression of *mf3* was repressed in both the wild-type strain and *perR* mutant (Figure 2A), suggesting that PerR may not directly participate in *mf3* regulation under oxidative conditions. Unlike *mf3* and *spd*, the expression of *sda1* under oxidative stress relies on the regulatory activity of PerR. Sda1, one of the most potent DNases in GAS, has been found to help GAS to escape from immune clearance by degrading neutrophil extracellular traps (NETs) [12,19]. These results suggest that GAS up-regulates *sda1* expression through sensing peroxide signals by PerR, which may help GAS to escape from the NETs.

In summary, our results showed that the peroxide responsive activity of PerR may directly contribute to GAS expressing *sda1* under oxidative stress. The molecular mechanism of the positive regulation of PerR on the *sda1* promoter remains for further studies.

## Supporting Information

Figure S1 **Growth curve of the wild-type (A20), *perR* mutant (SW612), and complementation strain (SW665).**
(TIFF)
