# Supplementary figures and images for: Peroxide Responsive Regulator PerR of group A Streptococcus Is Required for the Expression of Phage-Associated DNase Sda1 under Oxidative Stress
Source: PLoS One. 2013 Dec 3;8(12):e81882. doi: 10.1371/journal.pone.0081882 (PMC3849366; doi:10.1371/journal.pone.0081882)

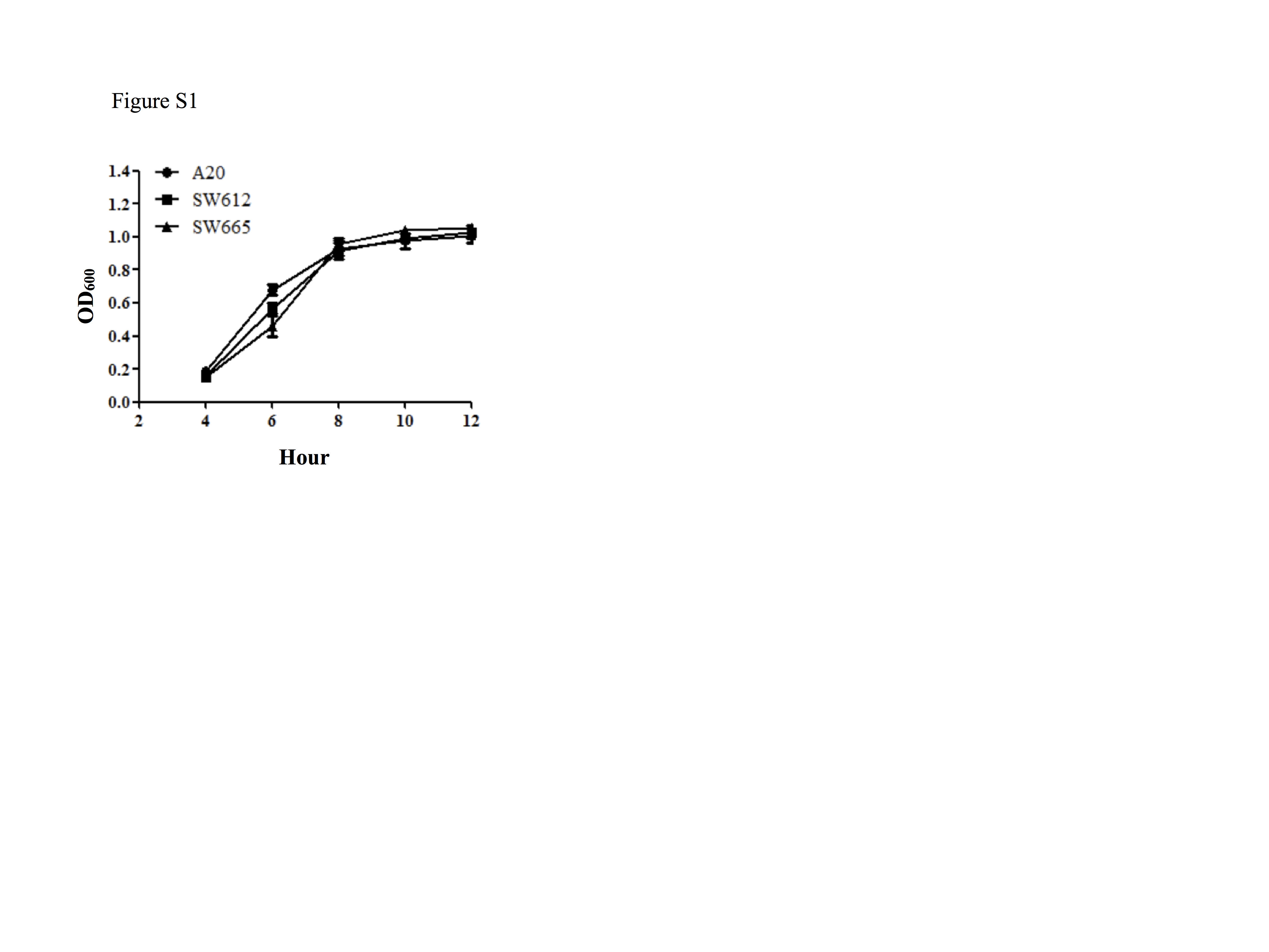

Supplement: Figure S1 — Growth curve of the wild-type (A20), perR mutant (SW612), and complementation strain (SW665). (TIFF) [file pone.0081882.s001.tiff]
